# Predictive Modeling of Pesticides Reproductive Toxicity in Earthworms Using Interpretable Machine-Learning Techniques on Imbalanced Data

**DOI:** 10.1021/acsomega.4c09719

**Published:** 2025-01-30

**Authors:** Mihkel Kotli, Geven Piir, Uko Maran

**Affiliations:** Institute of Chemistry, University of Tartu, Ravila 14a, Tartu 50411, Estonia

## Abstract

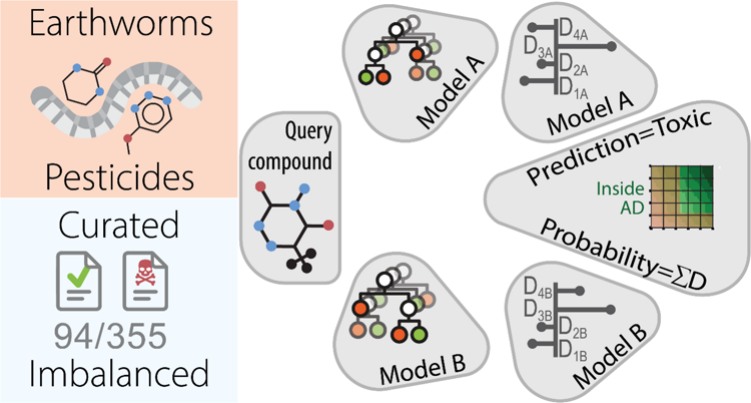

The earthworm is a key indicator species in soil ecosystems. This makes the reproductive toxicity of chemical compounds to earthworms a desired property of determination and makes computational models necessary for descriptive and predictive purposes. Thus, the aim was to develop an advanced Quantitative Structure–Activity Relationship modeling approach for this complex property with imbalanced data. The approach integrated gradient-boosted decision trees as classifiers with a genetic algorithm for feature selection and Bayesian optimization for hyperparameter tuning. An additional goal was to analyze and interpret, using SHAP values, the structural features encoded by the molecular descriptors that contribute to pesticide toxicity and nontoxicity, the most notable of which are solvation entropy and a number of hydrolyzable bonds. The final model was constructed as a stacked ensemble of models and combined the strengths of the individual models. Evaluation of this model with an external test set of 147 compounds demonstrated a well-defined applicability domain and sufficient predictive capabilities with a Balanced Accuracy of 77%. The model representation follows FAIR principles and is available on QsarDB.org.

## Introduction

Over the past century, pesticide utilization has dramatically evolved, reshaping agricultural practices, and profoundly impacting ecosystems worldwide. The dawn of synthetic pesticides in the early 20th century marked a paradigm shift in pest control, offering unprecedented efficacy and scalability over those of traditional methods. Increased use of pesticides due to the intensification of agriculture and the demands of a growing global population has yielded remarkable increases in crop yields and food security. However, this spread has raised significant concerns regarding environmental and health ramifications. From the introduction of dichlorodiphenyltrichloroethane (DDT) in the 1940s to the contemporary debates surrounding neonicotinoids, the journey of pesticide usage encapsulates a complex narrative of scientific innovation, regulatory challenges, and societal impacts. In the complexities of sustainable agriculture in the 21st century, understanding the multifaceted implications of pesticide use, including, but not limited to reproductive toxicity of pesticides, is imperative for devising informed policies and practices.^1,2^

Good soil is arguably the most critical element for successful farming. The presence of earthworms in the soil has a measurable effect on soil properties such as pH, texture, porosity, and organic matter content that benefit upregulated soil fertility and plant growth.^3^ They also play pivotal roles in forming soil structure, nutrient cycling, and decomposing organic matter.^4^ Therefore, the health of earthworms cannot be neglected when farming. Another essential factor to consider in successful farming is pest control. Pesticides are necessary tools in modern agriculture and have significantly contributed to the global increase in food production, but their widespread usage has raised concerns about environmental toxicity.^5^ And their unintended effects on nontarget organisms have become increasingly evident.

Earthworms are critical organisms in soil ecosystems and are vital indicators of soil health and ecosystem functioning.^4^ Therefore, the ecological and physiological features of the earthworms make them excellent indicators of soil pollution.^3^ Mortality or acute toxicity has been a commonly used criterion for assessing xenobiotics.^6^ However, it is suggested that for risk assessment, the earthworm reproductive toxicity should be the preferred property.^7^ Pesticides encompass a diverse array of chemical compounds that are designed to control pests. Unfortunately, most pesticides have shown some reproductive toxicity effects for earthworms.^8,9^ Even at low concentrations, pesticides have multiple adverse effects on earthworms, impacting growth, reproduction, behavior, enzymes, and DNA.^10^ For example, organophosphates and carbamates that inhibit acetylcholinesterase activity in earthworms, causing neurotoxic effects and reduced locomotor activity, are considered the most toxic.^11−13^ Therefore, chronic pesticidal exposure can profoundly impact earthworm populations, potentially disrupting soil processes and ecosystem services.^14^

Assessment of environmental risks caused by pesticides is inevitable. While experimental measurements provide the most reliable indications of risks, they are unnecessary in some scenarios.^15,16^ Using retrospective data-driven analysis and chemical structure-based modeling can be a cost-effective alternative for preliminary estimation of chemical behavior in the environment. Quantitative structure–activity relationships (QSARs) are a data-driven cheminformatic approach for both statistical description of the relationship and discovery of causal relationships between chemical cause and effect, in which the authors of the article have long-standing experience, including with modeling properties in toxicology.^17−30^ Computational toxicology protocols have become crucial for regulatory decision-making across various industries and regulatory bodies.^31^ As much as linear and multilinear models are more desirable in every thinkable manner for decision-makers due to such models being more interpretable and transparent, exhibited nonlinear relationships in complex biological systems require appropriate tooling.^32^ For example, Gradient-Boosted Trees (GBT) have gained significant popularity in various fields, particularly in building machine-learning based QSARs.^33,34^ However, interpretation and explainability of such machine-learning models is an open question in research.^35,36^

Modeling the chronic toxicity of pesticides in earthworms has challenged many researchers. Zhao and Xu have investigated neonicotinoids and their functional derivatives’ acute and chronic toxicity in earthworms with comparative molecular field analysis.^37,38^ Wang et al. studied the general biotoxicity of amide herbicides for ecotoxicological risk assessment across several organisms, including earthworms.^39^ Although not directly related to biological toxicity, the sorption behavior of chemicals in natural soil systems plays an important role concerning the observed toxic effects of pesticides on earthworms, as modeled by Binetin et al.^40^ with QSAR methods. Chlorophenols, chlorobenzenes, and chloroanilines are frequent scaffolds in some pesticide groups, and work has been done to investigate the toxicity and soil absorption simultaneously for such chemicals.^41^ As for toxicity models for heterogeneous sets of pesticides, there are acute earthworm toxicity models for pesticide compounds, e.g., a partial least-squares regression model^42^ and a linear discriminant analysis classification model.^43^ Also, our earlier work focused on a random forest classification model for pesticides’ acute lethality in earthworms.^6^ Based on the available literature and to the best of our knowledge, there appear to be no previous attempts to model a heterogeneous data set of pesticides and their chronic reproductive toxicity on earthworms.

The above provides a basis for the hypothesis that in data sets of structurally diverse pesticides, there is a relationship between structure and reproductive toxicity, and this can be modeled using machine-learning methods. To test this hypothesis, the first goal was to gather and create a data set that includes pesticide toxicity on earthworms’ reproductive ability and then use this data set to construct a stringently validated model based on chemical structure properties to predict the toxicity of unknown pesticide compounds in earthworms. The purpose of this work is to provide an initial assessment tool for regulatory decision-making regarding the fate of existing and newly registered compounds in agriculture and to discover insights into the biological and chemical mechanisms that control the metabolic fate of xenobiotics in earthworms.

## Methods

### Data Gathering and Curation

Raw data for toxicity was gathered from the Pesticides Properties Database^44^ as reproductive no-observed-effect concentration (NOEC). NOEC for earthworms is expressed as milligrams of compound in a kilogram of soil required to induce a statistical difference in the number of healthy juveniles produced after 4 weeks of reproduction followed by 4 weeks of monitoring.^45^

The initial data set contained 521 compounds from different pesticide classes like herbicides, fungicides, insecticides, and their metabolites. Unfortunately, some of them did not have all of the structural identifiers present. Therefore, PubChem and NCI databases were queried using any present structural identifier to gather missing information and perform structural curation. Structures with more than two distinct matching identifiers were considered valid if their Jaccard indices generated from Morgan6 fingerprints coincided. Structures with nonmatching structural identifiers (e.g., the SMILES and IUPAC names did not agree with each other) were manually examined to resolve syntactic errors and discarded otherwise. This reduced the size of the data set from 521 compounds to 482.

A fit-for-modeling data set with 464 compounds was then prepared by protonating salts and removing mixtures and organometallics. The gathered structures were standardized according to the workflow by Greg Landrum.^46^ Standardization is necessary to depict the same chemical functional groups in molecules in a homogenized manner within SMILES notation.

NOEC values of these compounds were converted to binary classes of toxic and nontoxic at breakpoint 100 mg/kg. This cutoff of 100 mg/kg is the boundary for the category of least harmful chemicals, recommended by the United Nations Committee of Experts.^47^ Since some of the NOEC values gathered were expressed as intervals instead of discrete values, they were converted as follows:

1. Real values <100 mg/kg to toxic class
2. Real values ≥100 mg/kg to nontoxic class
3. Intervals >0··· ≥ 10 mg/kg to toxic class
4. Intervals >10··· ≥ 100 mg/kg were discarded
5. Intervals >100 mg/kg to nontoxic class
6. Intervals <100 mg/kg to toxic class
7. Intervals <100···<inf mg/kg were discarded

**Figure 1:**
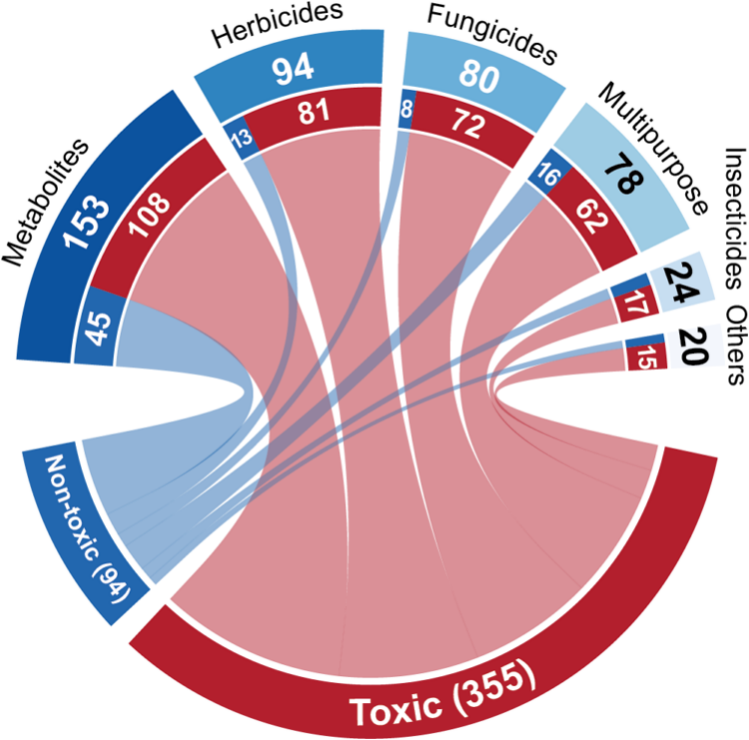
Distribution of pesticide classes and their reproductive toxicities in the QSAR-ready data set.

After class assignment, 449 QSAR-ready compounds remained in the data set, of which 355 were toxic and 94 were nontoxic (Figure 1). 147 compounds were set aside as an external validation set (Test set) via random sampling. 302 compounds were used to create the model.

### Chemical Structure Characterization

2199 2D molecular descriptors were calculated from standardized structures with Dragon 6.0.40^48^ and XLOGP 3.2.2.^49^ The exact definitions of the descriptors used in the analysis of the models originate from the Handbook of Molecular Descriptors.^50^

### Model Building–Tree-Based Algorithms

A decision tree is one of the simplest machine-learning models. Although they have some deficiencies, they are still the building blocks of tree-based models with cutting-edge performance for various tasks involving tabular data, e.g., QSARs.^34^ The simple modeling methods usually used first are linear discriminant analysis and logistic regression. However, initial modeling experiments with logistic regression (average BalAcc = 0.53) showed that more sophisticated modeling methods are needed to obtain more accurate results. Bagging and boosting are the two commonly used methods for developing tree-based ensemble models for QSAR; bagging is used by Random Forests and boosting is used by Gradient-Boosted Trees. Both algorithms (for more details, see SI) use different ensemble generation principles but generate many decision trees from the same input data.^6,27,51,52^

Bagged trees are numerous decision trees constructed from the same set of data, where some of the information has been purposefully removed. A sizable fraction of compounds and descriptors are concealed for each tree. By repeating this for hundreds of trees, the combined prediction of reduced trees displays better generalizability than one large tree trained on all data.^53,54^

**Figure 2:**
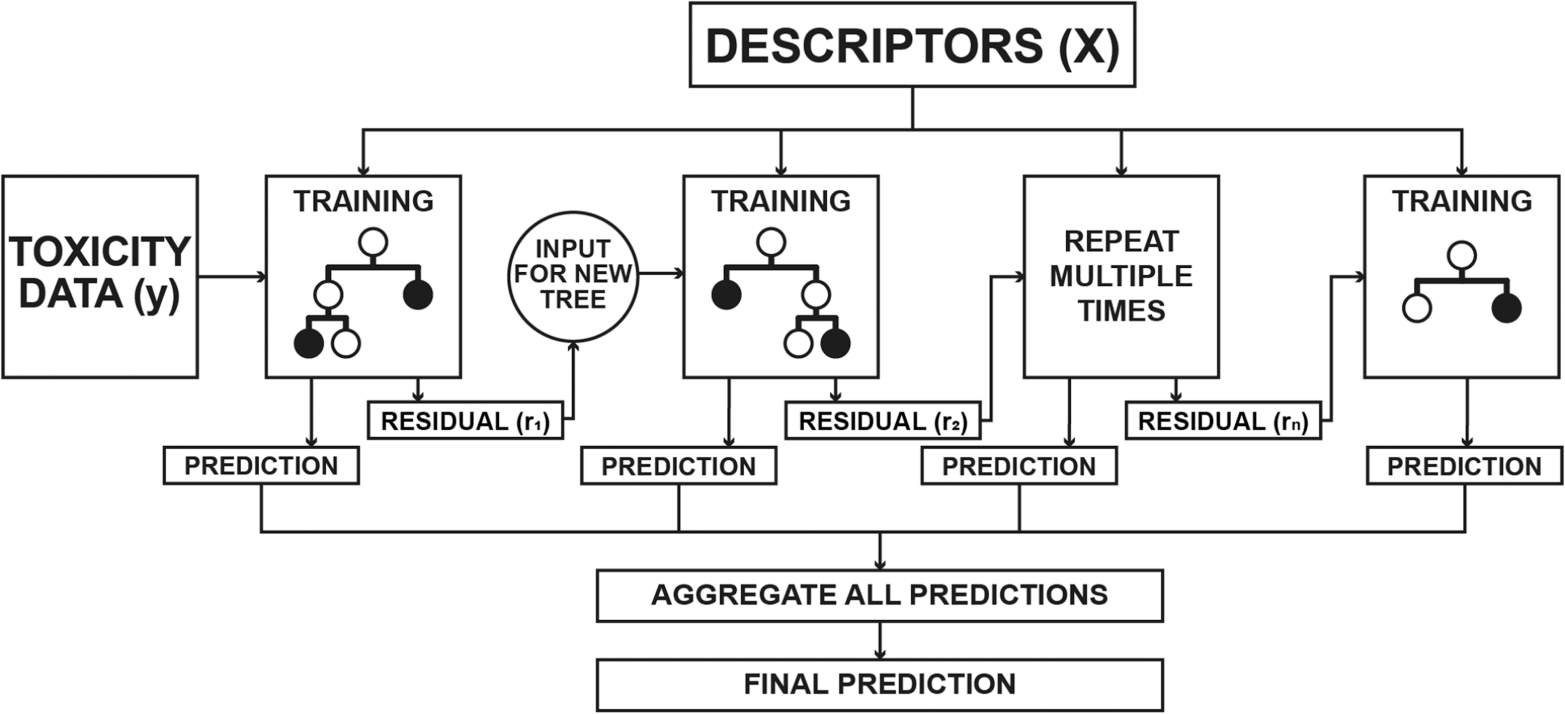
A stochastic gradient-boosted tree principle for regression. The prediction is composed of multiple trees (N trees). The first tree is constructed from available training data and label pairs (*X*, *y*). A fraction of data is set aside by random subsampling to reduce overfitting (*X*_sampled_, *y*_sampled_). Then, the first shallow decision tree, a weak learner, is built. Subsampling is also done for features, where at each split (decision node) in the decision tree, only a partial subset of features (descriptors) is considered for the splitting descriptor. After building the weak learner from subsampled data, it is evaluated on the whole training set by comparing predictions from the model to true labels (*r*_1_ = *y*–*ŷ*). The second weak learner in the ensemble is constructed in the same manner with a single difference that instead of predicting y-values, the error of the previous tree is predicted instead. Trees are added to the ensemble, until no further improvement can be made.

Boosted trees also involve the creation of several different decision trees to describe the same data set but in a different manner. For the case of bagged trees, the trees in the ensemble are independent of each other; i.e., the creation of the second tree is not affected by how the first tree was created. For boosted trees (Figure 2), the process involves creating a small decision tree, purposefully limited by size to create a weak learner for predicting *y* (toxicity) from *X* (descriptors). Then, the residuals (errors) of the first tree *r1* are used as the target, i.e., the second small tree is constructed to predict *r1* from *X.* This improvement-of-errors is repeated until convergence.^55,56^

### Performance Metrics–Model Evaluation

Several metrics (Table 1) are used to describe and compare the performance of the models. Most of those metrics are based on the number of true positive (TP), true negative (TN), false positive (FP), and false negative (FN) predictions. In this study, positive predictions correspond to the toxic and negative predictions to the nontoxic compounds.

**Table 1:** Performance Metrics and Their Range Used during and after Model Creation and Analysis^a^

abbr.	definition (range)	formula
TP	number of true positives	NA
TN	number of true negatives	NA
FP	number of false positives	NA
FN	number of false negatives	NA
Acc	accuracy (0···1)	
Sens	sensitivity (0···1)	
Spec	specificity (0···1)	
BalAcc	balanced accuracy (0···1)	
MCC	Matthew’s correlation coefficient (−1··· + 1)	
BS	Brier’s score (0···1)	

a In BS formula *C* is the set of all compounds, *c* is one compound, *y*_c_ is the true probability, Pr(*ŷ*_c_) is the predicted probability.

Accuracy (Acc) shows the overall predictive power of the model but can be deceptive in imbalanced data sets like the one used in this work. It might suggest high performance when the model predominantly predicts the majority (toxic) class. The Sensitivity (Sens) shows the proportion of correctly classified toxic compounds, and the Specificity (Spec) is critical for evaluating the model’s effectiveness on the nontoxic class. Accuracy calculated for an imbalanced data set may be too optimiztic. Therefore, Balanced Accuracy (BalAcc) was also used as a less biased metric for such data sets. It is calculated by averaging the Sensitivity and Specificity. Those metrics are used to evaluate the performance of final models.

Two other metrics were used, mainly for model optimization. Matthew’s Correlation Coefficient (MCC) is a more reliable metric in imbalanced settings as it balances the contributions of all confusion matrix elements, providing a holistic view of performance. Brier’s Score (BS) offers insights into the quality of probabilistic predictions, ensuring that the model’s confidence aligns with actual outcomes.

When constructing a QSAR, chance correlations may exist between the input descriptors and predicted property. Y-scrambling helps to assess whether the obtained QSAR is not created by chance. It is performed by creating a new synthetic data set where the relationship between descriptors and property is broken via randomizing (shuffling) the y-values. Then, a new model is created using the synthetic data set, and its performance metrics are compared against those of the original model. If the performance metrics are similar, it suggests that the original model may not be capturing a real relationship, and the apparent predictive power might have occurred by chance.^57^

### Bayesian Optimization–Guessing Hyperparameters

The aforementioned tree boosting and tree bagging algorithms have several parameters (called hyperparameters) that can be freely changed. Those changes can significantly influence the performance and complexity of the model. In contrast with parameters, they cannot be learned automatically from the data. Such hyperparameters are the shrinkage factor, the number of trees, the fraction of descriptors used at each split, and the fraction of compounds used at each tree. Finding optimal values for hyperparameters often means retraining the model with a given set of hyperparameters. Bayesian optimization is a way of guessing the best hyperparameters in an educated fashion, where the model’s output is used as a function of hyperparameters instead of compound-descriptor data. Different sets of hyperparameters are iteratively tested, and every next test (i.e., model retraining) performed with hyperparameters is believed to yield the most significant increase in the overall model performance.

### Genetic Algorithms—Descriptor Selection

Genetic algorithms are a way of solving optimization problems. The optimization problem at hand is descriptor selection using the smallest number of the most informative descriptors for building the underlying classifier, a tree-based model. Although tree-based models can perform inference adequately with random, noisy, or noninformative descriptors, using only relevant descriptors increases model performance, simplifying interpretation and usability. Individually trying out all of the combinations is not feasible in the current state of computing performance, as the number of possible descriptor combinations is larger than 10^6300^. Even at the optimiztic rate of one millisecond per combination and using all of the computing power available to mankind, it would still run into the heat death of the universe.

Thus, more clever approaches are needed. One way of describing different sets of descriptors is via binary encoding; i.e., for 2199 descriptors, a series with the same length of 0s and 1s is created. The resulting vectors can then be used in a process akin to evolution, where each distinct combination represents an individual with particular properties (a property is how well that descriptor set can describe chronic toxicity in earthworms). Different individuals are tested at each generation by changing them with genetic operators. The mutation operator promotes the exploration of search space, while the crossover operator promotes the exploitation of already known well-performing descriptors as candidates for the next generation.

In each generation, 50% of individuals participated in crossover, 10% in mutation, and 40% were left unchanged in a mutually exclusive manner. A pair of individuals exchanged descriptors randomly with a probability of . The chance of a mutation to occur in an individual chosen for mutation was .

242 toxic compounds and 60 nontoxic compounds were randomly split for every candidate descriptor set into a training set containing 80% of the compounds and a validation set containing 20%. After every generation, the training and validation sets were resampled. This produced imbalanced data sets. Under-sampling has shown better performance than oversampling for improving classifier performance for the minority class.^58,59^ Different oversampling strategies were also tried, but they yielded consistently weaker results than under-sampling. Based on previous research and experience, the training set was randomly under-sampled before fitting the model parameters to training data to attribute equal weightings to both the minority (*nontoxic)* class and the majority (*toxic)* class. The data set partitioning scheme is shown in Figure S1.

The hyperparameters pertaining to an individual’s set of descriptors were optimized after applying genetic operators. A Bayesian parameter search with 32 iterations was performed by 5-fold cross-validation on the training set. The hyperparameter set obtained after the search was kept as the best set pertaining to that individual’s combination. After optimization, the individuals continued to the evaluation stage. The search space, final hyperparameter values, and out-of-fold validation results are specified in Tables S1 and S2.

Various performance metrics were considered at each generation to decide whether an individual should continue to perform in the next generation. The Brier Score Loss and Matthew’s Correlation Coefficient were calculated for both training and validation sets, along with a penalty function  which is a monotonically decreasing function to apply evolutionary pressure toward selecting descriptor sets with fewer descriptors. Briar Score Loss was used to create a classifier that was calibrated as well as possible, whereas Matthew’s Correlation Coefficient was used to maximize trueness. For each model, five metrics were calculated MCC_train_, MCC_val_, BS_train_, BS_val_, and *P*(|*D*_*i*_|). The individuals carrying over to the next generation were selected based on those metrics with the NDSGA-II algorithm,^60^ which considers how well an individual performed for each metric and how similar it is to all others. This preserves the maximum genetic diversity while selecting the best possible individuals.

### SHAP—Descriptor Contributions

Given the intrinsic ability of tree-based models to model nonlinear relationships compounded with the complexity arising from ensemble methods, the resulting prediction can be opaque with respect to original input descriptors. Several model interpretation methods have been developed in the past decade to remedy this conundrum. Feature permutation importances,^53^ Local Interpretable Model-agnostic Interpretations (LIME),^61^ and Shapley values^62^ are well-known examples of such methods. The first two have their advantages, but compared with Shapley values, they lack the necessary qualities such as globality or directionality. Therefore, Shapley values were used as an explanatory model for more complex models.

This means that a linear model (eq 1) can be used as an explanatory model to describe and analyze the more complex tree-based model.^62,63^
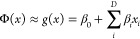
1The coefficients β directly show the contribution of each descriptor to a prediction. They are calculated by looking at sums over subsets (eq 2), where one or more descriptors are removed from the model for each subset.
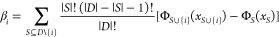
2L.S. Shapley formalized this approach, and the importance coefficients derived from the idea above are called Shapley values.^64^ A similar but computationally more feasible formulation was proposed by Lundberg et al., which they named SHAP values. A compound’s descriptor vector *x* can be mapped to a coalition vector *x*′ via binary encoding. *x*′ is a complete coalition, a vector of ones. Instead of a complete coalition, an incomplete coalition *z*′ can be formed, e.g., *z*′ = (1,1,0,1). Mapping back to the feature space by *h*_*x*_: *z*′ → *z* produces an altered training instance, where 1’s are mapped to the original feature value of *x*, but 0s are stochastically mapped to descriptor values sampled from the same distribution as the data set, i.e., replaced with the same descriptor value of another compound from the data set.

To properly weigh those modified instances, the SHAP kernel π_*x*_(*z*′) is introduced to attribute more weight to coalitions in which only one descriptor is present or missing. This means the difference between the original and explanatory models’ outputs is minimized via L2 loss, and SHAP values are the result of following minimization procedure
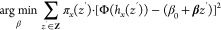
3where, **β** is a vector of SHAP values, ***Z*** is the set of all altered training instances, and β_0_ is the baseline value.

### Ensembling Models—Constructing Applicability Domain

Ensembling methods for applicability domain (AD) estimation typically involve aggregating predictions from different QSAR models and evaluating the consensus. This can be done by using bagging, boosting, and stacking techniques. Bagging (Bootstrap Aggregating) generates multiple versions of a model by training on different subsets of data and then averaging the predictions. Boosting sequentially applies models to the residuals of previous models to improve accuracy. Stacking combines predictions from several models using a meta-model to determine the final prediction.^65,66^

Recent studies have explored the use of stacked classifiers in QSAR modeling for toxicity prediction. Using a stacked ensemble classifier for predicting HCV NS5B inhibitors, it was possible to achieve an accuracy of over 85%.^67^ On ToxCast data, stacked generalization outperformed simple QSAR models and improved performance for 61% of 483 models.^68^ A stacked ensemble of machine-learning methods to predict fish toxicity achieved better prediction accuracy compared to existing tools.^69^ Also, the integration of different regression and classification models for acute oral systemic toxicity prediction outperformed all base models.^70^ These studies highlight the potential of stacked classifiers to enhance the QSAR model performance in various toxicity prediction tasks.

Furthermore, comparing or combining predictions across different models with different input parameters is one of the most reliable methods to estimate AD.^71^ This can significantly improve the model quality and introduce the applicability domain via thresholding rules. This study uses a similar AD approach by aggregating prediction probabilities and searching optimal thresholds for AD. Models in this study output probability in the range of 0.0 to 1.0; a probability above 0.5 means the compound is classified as toxic, and a probability below 0.5 means the compound is nontoxic. In an ensemble prediction, the prediction probabilities of several models are combined in an additive way and normalized to 0 by subtracting the thresholds of the individual models. Thus, for the ensembled prediction, a prediction probability above 0 means the compound is toxic and below 0 is nontoxic.

The threshold for classifying a compound from prediction probability to a particular class can be adjusted depending on the necessary strictness. Increasing the threshold required to classify a compound as toxic and decreasing the threshold required to classify a compound as nontoxic will constrict the coverage of the ensembled model. The Coverage, the ratio between predictable compounds to all compounds, determines how many compounds are inside the applicability domain of the ensembled model.

## Results and Discussion

### Performance of the Models and Estimation of the Applicability Domain

Hundreds of models with varying characteristics were generated as part of the evolutionary algorithm. In addition to overall performance, Model A was selected as the best in terms of Specificity, and Model B was selected in terms of Sensitivity. Y-scrambling was performed 100 times for both models, and results showed that neither model was produced due to chance.

The performance of two models, A and B, was analyzed using various metrics (Table 2). Usually, the imbalance of the data set presents a challenge in evaluating the quality of the models. However, in the current case, all overall performance metrics (Table 2: Acc, BalAcc, and MCC) showed comparable results for all of the models.

**Table 2:** Performance Metrics of Models A and B

model	set	Sens	Spec	Acc	BalAcc	MCC	TP	TN	FP	FN	total
A	train(A)	0.64	1.00	0.71	0.82	0.50	124	46	0	71	241
A	val(A)	0.62	0.79	0.66	0.70	0.34	29	11	3	18	61
A	test	0.57	0.79	0.62	0.68	0.30	64	27	7	49	147
B	train(B)	0.84	1.00	0.88	0.92	0.73	160	51	0	30	241
B	val(B)	0.67	0.67	0.67	0.67	0.25	35	6	3	17	61
B	test	0.72	0.59	0.69	0.65	0.27	81	20	14	32	147

Model A demonstrates higher specificity (nontoxic class is well predicted). It maintains relatively consistent performance across the data sets, correctly predicting 79% of nontoxic compounds in validation and test sets (Table 2). However, Model A’s sensitivity is lower, particularly in the test set (0.57), suggesting it misclassifies more toxic cases as nontoxic (FN predictions). Nevertheless, its overall accuracy and balanced accuracy for validation and test set were between 0.62 and 0.7. This model might be suitable for occasions where maximizing true negatives is a priority (i.e., lead compound development).

In contrast, Model B exhibits higher sensitivity, which implies better identification of toxic cases but at the expense of increased false positives (FP), as reflected in its lower specificity in the validation (Table 2: 0.67) and test sets (0.59). Model B is more aggressive in identifying toxic compounds than Model A. Unfortunately, it also leads to more FP predictions. Being better at identifying the majority class (toxic) also gives better accuracy for the test set (0.69), but in general, accuracy and balanced accuracy were within the range of Model A. The use case of this model might be more in environmental risk assessment, where the requirement is to minimize false negatives.

From the point of view of MCC, Model A has better generalization power than Model B. Model A respective Training set metrics compared with Validation and Test set metrics (Table 2: 0.50 vs 0.34 and 0.50 vs 0.30) have less difference than respective Model B metrics (Table 2: 0.73 vs 0.25 and 0.73 vs 0.27).

**Figure 3:**
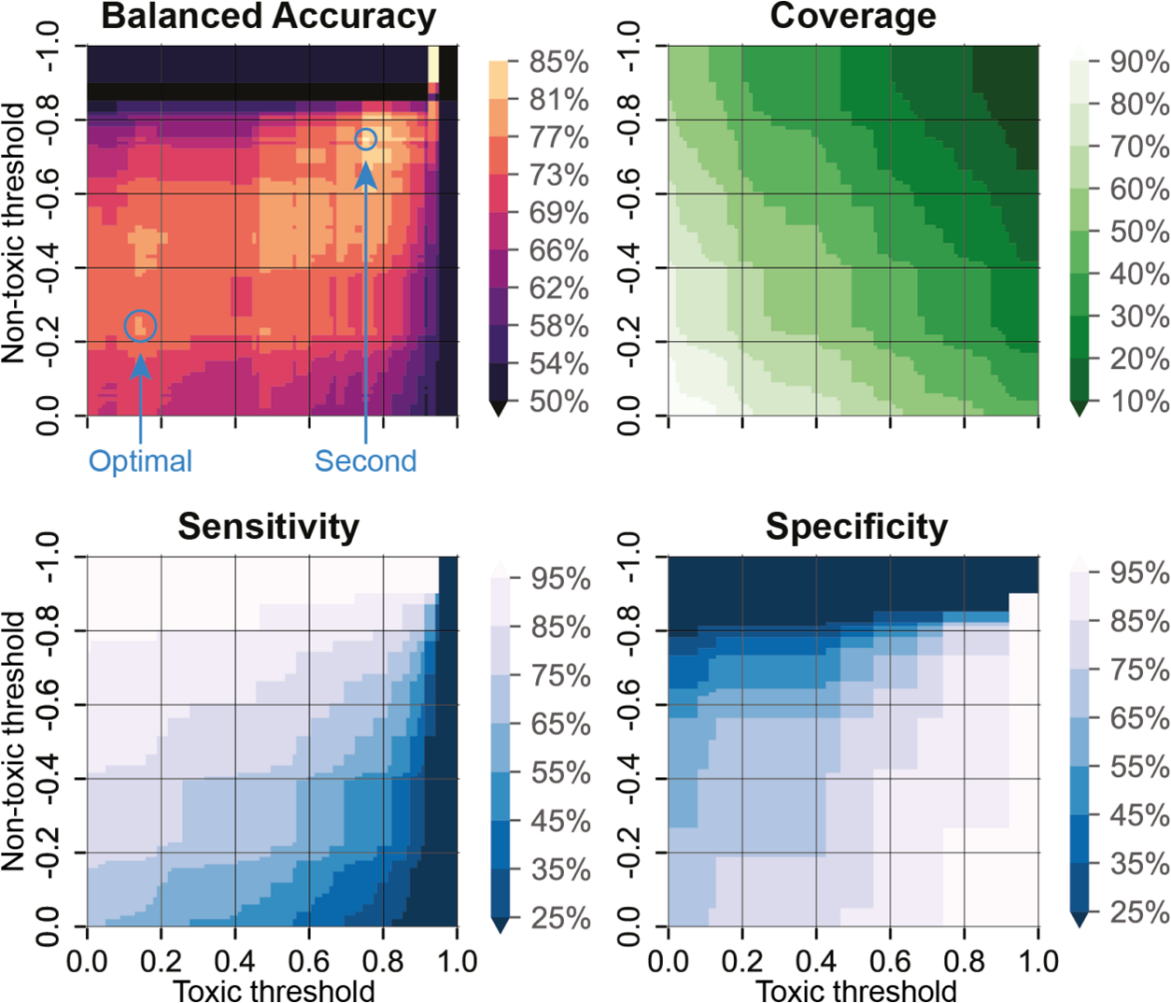
Effect of prediction probability thresholding on coverage and performance metrics was evaluated on the test set, where thresholds were constricted at two ends. For example, setting Toxic threshold = 0.2 and Nontoxic threshold = −0.4 restricts the model to predictions, where a compound is classified as toxic if the sum of SHAP values from both models exceeds 0.2 and classified as nontoxic if the sum of SHAP values from both models is below −0.4. According to the figure, the Sensitivity of the ensemble at that threshold is in the range of 65 to 75%, and the Specificity of the ensemble is between 65 and 75%. The optimal threshold is at (Toxic = 0.14; Nontoxic = −0.23), which yields 77% Balanced Accuracy on the test set at a coverage of 73%. The second threshold (Toxic = 0.75; Nontoxic = −0.75) yields a Balanced Accuracy of 85% at a coverage of 18%. The two regions are significantly different regarding their balanced prediction accuracies but vastly different regarding their coverage. The second threshold is quite the upper limit in terms of usability for a balanced prediction at the expense of coverage.

To improve prediction quality, Models A and B were ensembled to produce a unified model (Model C). Model C is a stacked ensemble prediction where the classification label (*toxic* or *nontoxic*) is assigned from the sum of the prediction probabilities of individual models. This combines the strengths of both models with the drawback of reduced coverage when different thresholds for toxicity and nontoxicity are applied. Variable thresholds across the range (Figure 3) and their effects on the Balanced Accuracy, Sensitivity, Specificity, and Coverage of the test set allow for model selection.

Analysis of those metrics suggested that relatively high coverage and good performance metrics are present at Toxic threshold = 0.14 and Nontoxic threshold = −0.23 (Figure 3). Table 3 presents the ensembled model’s performance metrics and coverage at this threshold. This threshold limits the available number of compounds to 107 out of 147, i.e., 73% of compounds are inside the Applicability Domain at that threshold. This yields around a 10% improvement in both Accuracy and Balanced Accuracy.

**Table 3:** Performance Metrics of the Ensemble Model with Constricted Applicability Domain (Toxic = 0.14, Nontoxic = −0.23)

model	set	Sens	Spec	Acc	BalAcc	MCC	TP	TN	FP	FN	total
A&B	train	0.81	1.00	0.85	0.90	0.69	106	39	0	25	170
A&B	val	0.80	0.50	0.75	0.65	0.30	8	1	1	2	12
A&B	test	0.80	0.74	0.79	0.77	0.52	64	20	7	16	107

As can be seen from the comparison of the total column from Tables 2 and3, the number of compounds available for evaluation for the ensemble model is reduced. This is due to two reasons. The first reason is an inherent mismatch of the two models—the sum of prediction probabilities is outside the required threshold. The second reason is present only in training and validation sets—the stochastic resampling of training and validation sets during the genetic process resulted in data sets with different compositions. Of the 241 compounds in training sets, 194 are shared, and of the 61 compounds in validation sets, 14 are shared.

### Descriptor Analysis

**Figure 4:**
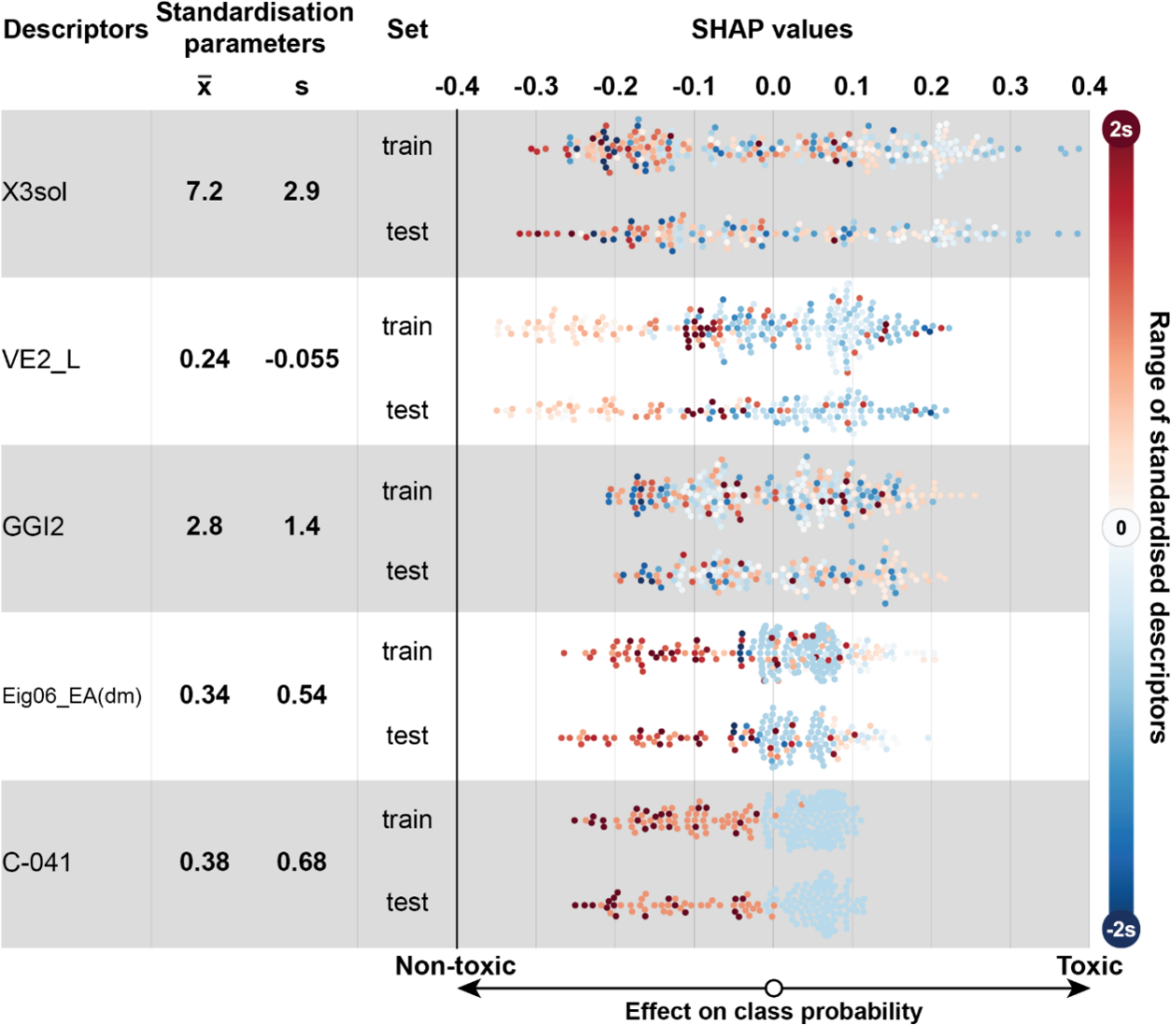
SHAP analysis for Model A with 5 descriptors. Each row corresponds to one descriptor in which compounds from both training and test sets are shown as circular markers. Each descriptor was standardized using their training set’s mean (x̅) and standard deviation (s). The horizontal position of each marker corresponds to its SHAP value. In contrast, its color is determined by the descriptor value of the compound (orange if it is higher than its mean and blue if it is lower than the mean). The figure shows how descriptor values affect the model’s prediction whether a compound is classified as toxic or nontoxic. For example, the mean value for C-041 is 0.38 and compounds with C-041 values above that value (orange) tend to be nontoxic as their SHAP values are negative. Furthermore, compounds with less than the mean value (blue) have a higher (positive SHAP values) likelihood of the compound being classified as toxic by the model. This applies to the training and test set compounds.

**Figure 5:**
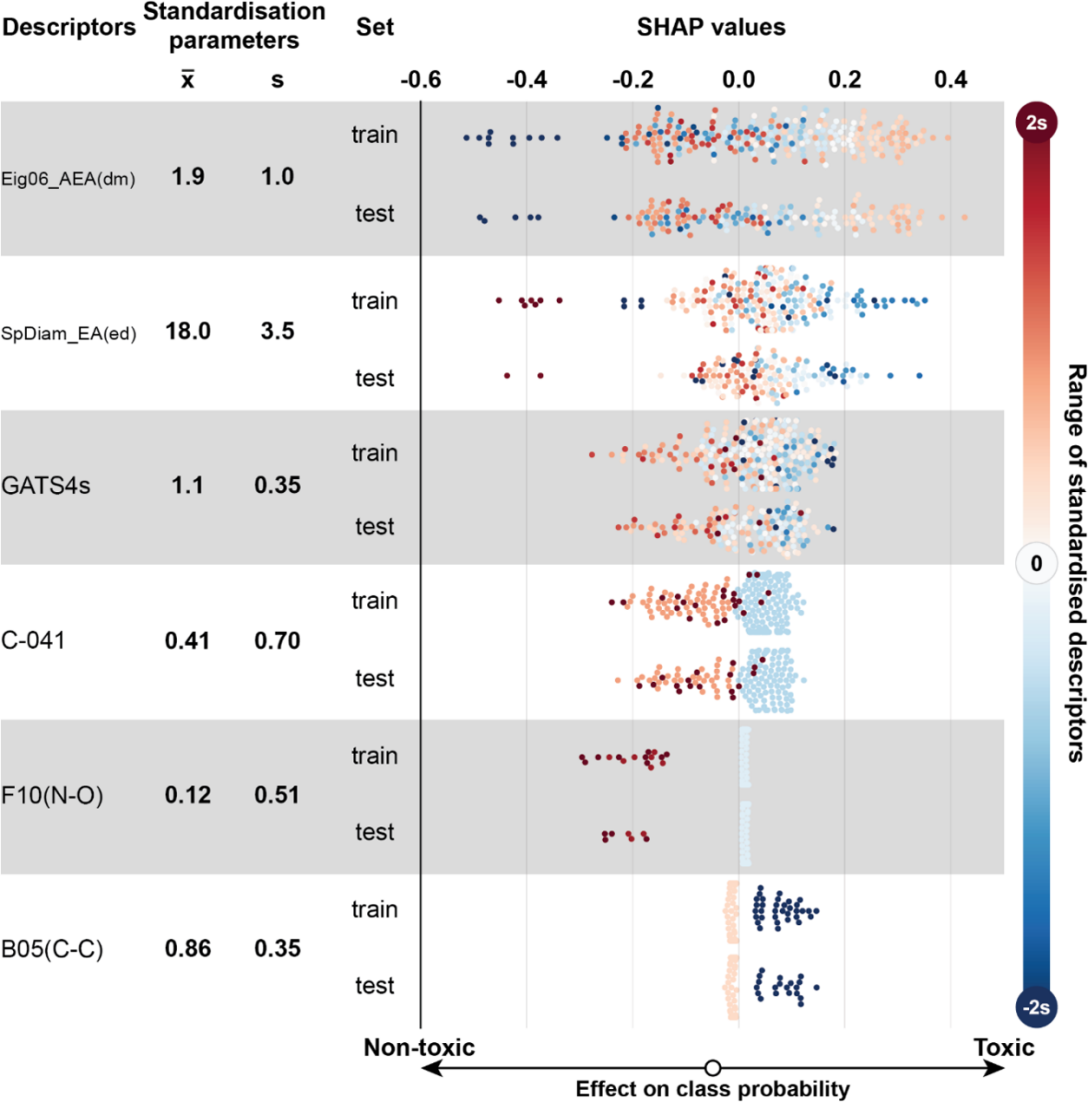
SHAP values of the external validation set (test) and training set (train) for Model B. Information about descriptors and their corresponding SHAP values are shown similarly to Figure 4.

**Figure 6:**
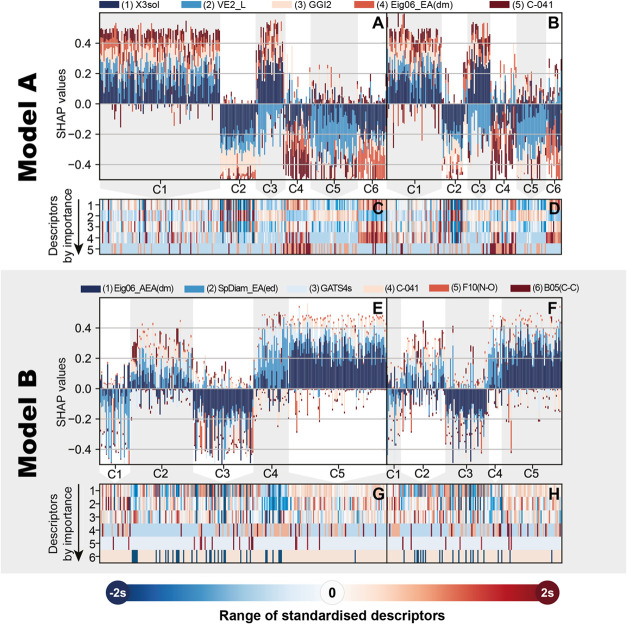
Clustering performed on training and test compounds SHAP values of Model A (A–D) and Model B (E, F, G, H). An alternative, more detailed view of SHAP and descriptor values from Figures 4 and 5 are shown for Models A and B, respectively. The SHAP values for training and test data sets are shown on subfigures A, B, E, and F. The descriptor values for training and test data sets are shown on subfigures C, D, G, and H. A Vertical slice within one model’s barplot is the same compound on the heatmap.

Understanding how each descriptor’s value shapes the final prediction is challenging when a model involves multiple decision trees. To better understand how the descriptor values affect the predictions of both models, SHAP analysis and clustering were performed on models and data sets. SHAP analysis gives descriptor importance (Table 4) and provides means for the visualization of descriptor contribution (Figure 4 for Model A, Figure 5 for Model B). Clustering of SHAP values and descriptor values allows one to understand how descriptors contribute to the grouping of compounds (Figure 6 summarizes both Model A and Model B).

**Table 4:** Descriptors and Their Importance According to SHAP Analysis for Both Models

ID	model	importance	name from DRAGON
X3sol	A	1	solvation connectivity index of order 3
VE2_L	A	2	average coefficient of the last eigenvector from Laplace matrix
GGI2	A	3	topological charge index of order 2
Eig06_EA(dm)	A	4	eigenvalue no. 6 from edge adjacency mat. weighted by dipole moment
C-041	A, B	5, 4	X–C(=X)–X
Eig06_AEA(dm)	B	1	eigenvalue no. 6 from augmented edge adjacency mat. weighted by dipole moment
SpDiam_EA(ed)	B	2	spectral diameter from edge adjacency mat. weighted by edge degree
GATS 4s	B	3	geary autocorrelation of lag 4 weighted by I-state
F10[N–O]	B	5	frequency of N–O at topological distance 10
B05[C–C]	B	6	presence/absence of C–C at topological distance 5

For Model A, all compounds from two data sets, training set compounds in Figure 6A,C, and test set compounds in Figure 6B,D are shown. Top row bar plots in Figure 6A,B show the SHAP values generated by the model for the same compounds. Bottom row heatmaps (Figure 6C,D) show the two data sets’ descriptor values for each compound. Compounds are ordered so that one vertical slice displays SHAP and descriptor values across two parts of the plot. For Model B, analogous plots are shown in Figure 6 subfigures E, F, G, and H.

#### Descriptors in Model A

Model A uses five molecular descriptors, and the most significant descriptor in Model A is X3sol (Figure 4), which is the solvation connectivity index of order 3 (for more details, see SI). Solvation connectivity indices model solvation entropy and describe dispersion interactions in a solution via quantum numbers of atoms in the molecule and the connectivity of said atoms.^72^ The effect of this descriptor on the probability of the compound being toxic is somewhat convoluted. It seems that extremely high values of this descriptor (>12) and extremely low values of this descriptor (<4) can be correlated with a compound having low reproductive toxicity (SHAP < −0.1), as can be seen in Figure 4. Such compounds were also clustered together (clusters C2, C4, and C6, dark blue), as seen in Figure 6. Compounds with a descriptor value around the mean (X3sol = 7.2) exhibit more chronic reproductive toxicity, as seen in clusters C1 and C3 (Figure 6). This descriptor is strongly proportional to the overall molecular weight, with polyfluorinated compounds being the main outliers (fluorine atoms are not included in the calculation).

The second most important descriptor was VE2_L, the average coefficient of the last eigenvector (absolute values) from the Laplace matrix. Based on SHAP analysis, compounds with above mean VE2_L (0.24) descriptor value are more likely nontoxic, and compounds with below mean descriptor value tend to be more toxic (Figure 4). Clustering suggests that this descriptor has the highest influence on compounds in cluster C5 (Figure 6, light blue).

The GGI2 is the topological charge index of order 2, effect of which in Model A is difficult to analyze. The SHAP values of this descriptor do not offer a discernible pattern (Figure 4). However, clustering shows that the highest influence of this descriptor is on cluster C2, where extremely small values for this descriptor seem to indicate nontoxicity. Compounds with low GGI2 SHAP (< −0.15) and descriptor (<1.5) values are low molecular weight adjuvants or solvents such as dichloropropene, ethephon, and chloroaniline.

Eig06_EA(dm) is calculated from an edge adjacency matrix, which describes the connectivity between atoms in a molecule. The value of this descriptor is 0 for many compounds in the data set. Despite this, it still provides valuable information in combination with other descriptors. Higher values of the Eig06_EA(dm) descriptor (>0.4) are correlated with lower reproductive toxicity (Figure 4), and it mainly affects compounds in clusters C4 and C6 (Figure 6).

C-041 is the number of esteric, carboxylic, amidic, and similar fragments where a carbon atom has one double bond and two single bonds to heteroatoms (X–C(=X)–X). High values of C-041 are directly related to a high occurrence of the corresponding functional group in a molecule, and analogously, low values of that descriptor point to a low occurrence of such functional groups. Many such compounds are located in cluster C4. The functional groups in question include molecules with ester and peptide bonds, which seems to increase the likelihood of the compound being classified as nontoxic (see Figure 4). In contrast, its absence increases the likelihood of being more toxic. Carbamates make up a significant individual class of insecticides containing such a functional group. Although many carbamates, such as IPC (isopropyl *N*-phenylcarbamate), are considered extremely toxic in the acute time frame in higher doses, they are not so in the chronic time frame with lower doses. As molecules are likely to be hydrolyzed as part of detoxification pathways, the lack of hydrolyzable groups may be related to increased toxicity in earthworms, or, instead, the existence of such groups allows the compound to be metabolized to less harmful metabolites.

#### Descriptors in Model B

Model B uses information from six descriptors (Figure 5, for descriptors’ theoretical background, see SI), and clustering of SHAP values revealed five distinct clusters (Figure 6). The two most significant descriptors in Model B are Eig06_AEA(dm) and SpDiam_EA(ed), which are both eigenvalue-related descriptors (Figure 5). Eig06_AEA(dm) is eigenvalue no. 6 from the augmented edge adjacency matrix weighted by dipole moment. Although Eig06_EA(dm) from Model A and Eig06_AEA(dm) from Model B seem similar in principle, they are constructed from different matrices and thus encode different information. Clustering shows (Figure 6) that this descriptor mainly influences compounds in clusters C3 and C5. Chronic toxicity correlates with the Eig06_AEA(dm) descriptor for positive values up to 2.7 (Figure S2), after which the model considers the compounds nontoxic.

SpDiam_EA(ed) is the difference between maximal and minimal eigenvalue from the edge adjacency matrix weighted by the edge degree. Chronic earthworm reproductive toxicity is affected by the eigenvalue descriptor SpDiam_EA(ed) in a seemingly inverse linear fashion where compounds with lower descriptor values are considered toxic and with higher values nontoxic (Figure 5). A closer investigation of clusters (Figure 6) reveals that the highest influence of this descriptor falls to clusters C1 and C4. The first contains many compounds with maximum values of this descriptor, and the second includes compounds with below-average values.

GATS 4s is Geary autocorrelation of lag 4 weighted by the I-state. It seems (Figure 5) that descriptor values above 1.1 contribute more to nontoxic prediction and below to toxic prediction.

Descriptor C-041 is the only one that appears in Models A and B. Its effect on toxicity prediction is the same in both models, i.e., the presence of X–C(=X)–X fragment indicates nontoxicity.

F10(N–O) is the number of nitrogen–oxygen atom pairs at a topological distance of 10. B05(C–C) is the presence or absence of any carbon–carbon atom pairs at topological distance 5. SHAP values of F10(N–O) indicate that the compound is more likely to be nontoxic (Figure 5). The explanation for this is related to molecule size rather than a specific interaction from pesticide and biotarget as the data set is too heterogeneous to be the cause of any particular interaction resulting from distance 10 topological placement of nitrogen and oxygen atoms. The argument is similar for B05(C–C) as the atom pair it considers is very general and present in most pesticide molecules, apart from relatively small molecules. The conclusion from the SHAP values of both descriptors above is that molecules with small sizes are more likely to have chronic reproductive toxicity in earthworms (Figure 5).

### Interpreting Model Outputs

**Figure 7:**
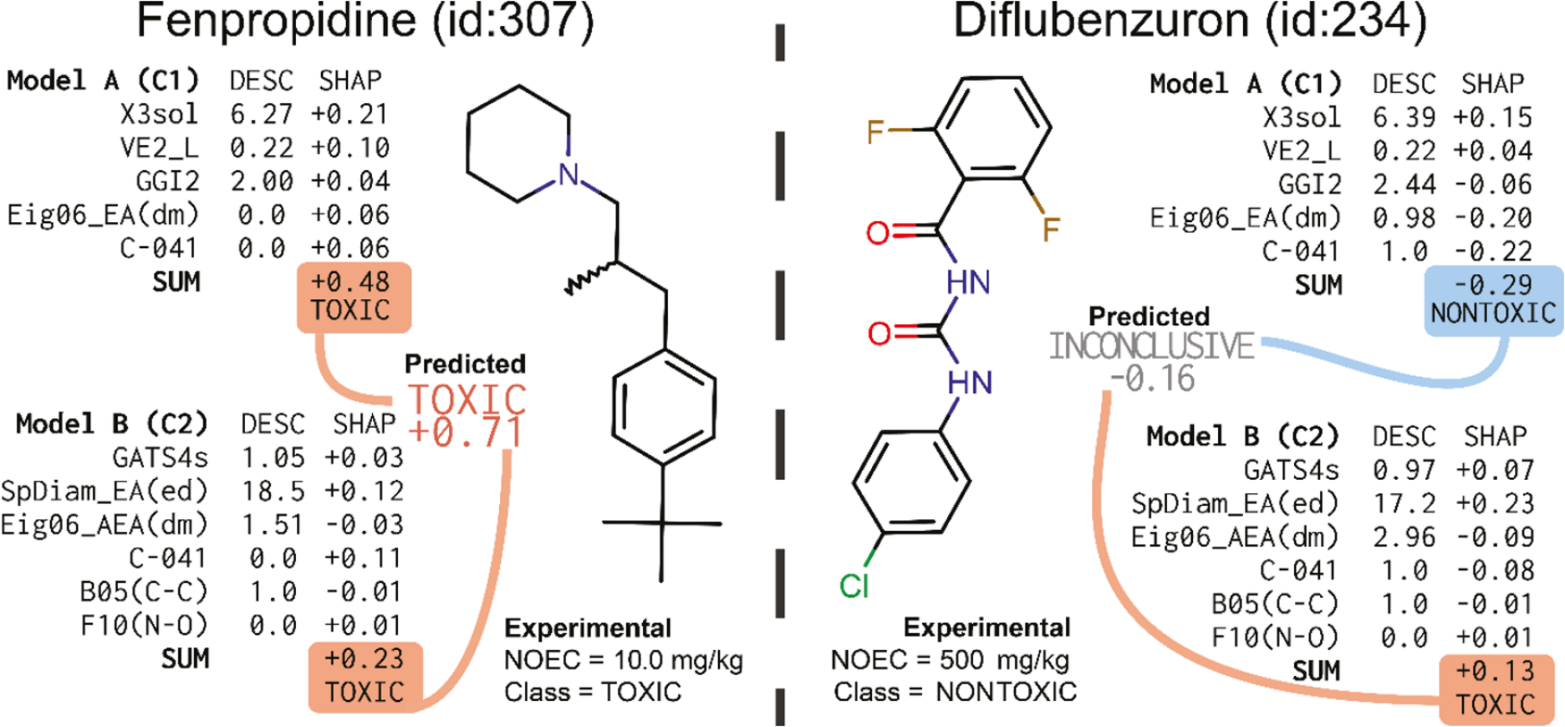
An example prediction for two fungicides (diflubenzuron and fenpropidine). The structure of the pesticide is entered, and the descriptors are calculated. Based on descriptors, the models output their predictions. The output is further analyzed with SHAP values to uncover which descriptors affected predictions the most. For Model A prediction, fenpropidine, the most important descriptor, is X3sol, which increases the likelihood of the compound being classified as toxic by 21%. The predictions from both models along the SHAP values are shown. Fenpropidine belongs to cluster C1 in Model A and C2 in Model B. The compound is classified as toxic; the sum of SHAP values from both models being 0.71 means that there is high confidence that the compound has chronic reproductive toxicity below the cutoff of 100 mg/kg.

As an example, an explanation of the output of the model(s) is provided for two different compounds: fenpropidin, a fungicide that inhibits sterol biosynthesis, and diflubenzuron, an insecticide disrupting chitin synthesis used for controlling moths (Figure 7). Predictions from both models are shown along with descriptor and SHAP values.

#### Fenpropidine

Models A and B both predict fenpropidine to be toxic. Interestingly, the more sensitive one (Model A) implies a higher probability of being toxic than Model B. The descriptor that most influences the prediction of Model A is X3sol. Still, other descriptors contribute to the likelihood of the compound being classified as toxic (all have positive SHAP values). SHAP contributions for fenpropidine in Model B vary, but two strong indicators, SpDiam_EA(ed) and C-041, have the most impactful contributions. The individual models and ensembled predictions (the sum of all SHAP values = 0.71) mean there is high confidence that the compound has chronic reproductive toxicity below the 100 mg/kg cutoff.

#### Diflubenzuron

In the case of diflubenzuron, two models yield disagreeing predictions. Model A, i.e., the more specific model, predicts the compound to be nontoxic, and Model B, the more sensitive model, predicts the compound to be toxic. Model A nontoxic prediction is mainly due to contributions from two descriptors, an eigenvalue descriptor Eig06_EA(dm) and a fragment descriptor C-041. For both of those descriptors, the corresponding SHAP value is negative. The descriptor value of Eig06_EA(dm) is very high (0.98), approximately 2 standard deviations above the mean (see Figure 4), which is in concordance with the general trend among the majority of compounds across all data sets (Figure 4). The eigenvalue descriptor contributes a negative amount (−0.20) to the probability of the compound being toxic, as indicated by its SHAP value (Figure 4). The C-041 descriptor has an even higher contribution to diflubenzuron being nontoxic, with the SHAP value of −0.22 from a single X–C(=O)–X fragment. In aggregate, the combined SHAP values from all descriptors in Model A sum to −0.29, rendering the final prediction to be *nontoxic*.

Model B prediction for diflubenzuron is that the compound is toxic, i.e., has NOEC value below 100 mg/kg. This prediction is entirely dominated by the SpDiam_EA(ed) descriptor. The descriptor value 17.2 for diflubenzuron is practically equal to the mean value across different data sets, according to Figure 5. Furthermore, the same figure shows that for the descriptor SpDiam_EA(ed), mean-valued compounds are distributed sporadically and do not clearly prefer either side of the decision boundary. Considering the whole model with its SHAP values, the final output of Model B is that difluorobenzuron is predicted to be toxic, albeit with weak confidence.

The ensemble prediction yields a combined SHAP value of −0.16. The suggested threshold for classifying a compound as nontoxic from Figure 3 is −0.23. Thus, the difluorobenzuron is outside of the suggested applicability domain.

## Conclusions

Chronic reproductive toxicity data of pesticides on earthworms as no-observed-effect-concentration (NOEC) were gathered from publicly accessible sources. A breakpoint of 100 mg/kg was established as the cutoff for binary classification of compounds as *toxic* and *nontoxic*. Different machine-learning methods and algorithms (gradient-boosted decision trees, genetic algorithm, and Bayesian optimization) were employed, improved, and implemented to create and select the best predictive model. Three models were derived and extensively validated as a result: a sensitivity-oriented model (0.72 Sens) for identifying toxic compounds, a specificity-oriented model (0.79 Spec) for identifying nontoxic compounds, and a balanced ensembled model (0.75 BalAcc) combining the strengths of previous two models at the expense of a reduced applicability domain. Analysis of models’ descriptors provided insights into how chemical structure determines possible mechanisms of toxicity and detoxification in the earthworm. The proposed classification models, descriptors, and experimental data can be accessed in the QsarDB repository^73,74^ according to FAIR principles (10.15152/QDB.263).

## Data Availability

Chemical structures were characterized with DRAGON 6.0.40 and XLOGP 3.2.2. Models were developed using scikit-learn 1.1.3. The model and related data are available in various formats. To follow the best practices of QSAR model reporting, the QSAR Data Bank format is used, and models with data are stored in the QsarDB repository. A digital object identifier (DOI) is assigned for the models and data (10.15152/QDB.263).

## Supporting Information Available

Detailed information about tree-based models and used descriptors; schema visualizing data set partitioning after curation and class assignment; correlation between Eig09_AEA(dm) SHAP and descriptor values; hyperparameter ranges and final values for Model A and Model B; cross-validation results in test fold on the training set during Bayesian optimization for Model A and Model B (PDF)
