# Supplementary material for: Predictive Modeling of Pesticides Reproductive Toxicity in Earthworms Using Interpretable Machine-Learning Techniques on Imbalanced Data
Source: ACS Omega. 2025 Jan 30;10(5):4732–44. doi: 10.1021/acsomega.4c09719 (PMC11822515; doi:10.1021/acsomega.4c09719)
Supplement: Supplementary file 1 — ao4c09719_si_001.pdf [file ao4c09719_si_001.pdf]

Predictive modelling of pesticides reproductive toxicity in earthworms using interpretable machine learning techniques on imbalanced data

Mihkel Kotli, Geven Piir*, Uko Maran

Institute of Chemistry, University of Tartu, Ravila 14a, Tartu 50411, Estonia

* Corresponding author:
e-mail: geven.piir@ut.ee
phone: +3727375278

Supplementary Information

Bagged and boosted trees

A decision tree is one of the simplest machine learning models. Although they have some deficiencies, they are still the building blocks of tree-based models with cutting-edge performance for various tasks involving tabular data, e.g., quantitative structure-activity relationships[1]. Commonly used ensemble models in QSAR are Random Forests and Gradient Boosted Trees, which generate many different decision trees from the same input data[2,3,4].

Given an input descriptor matrix $\mathbf{X}^{C \times D}$ of D descriptors for C compounds and known (true) experimental values of the property $\mathbf{Y}^{C \times 1}$, an individual trained decision tree T maps compounds from descriptor space to property space $T : \mathbf{X} \mapsto \widehat{\mathbf{Y}}$. The error between actual and predicted values $(\mathbf{Y} - \widehat{\mathbf{Y}})$ can be used to evaluate the final model's quality via performance metrics and during iterative model generation via loss functions[5].

In the case of bagged trees, numerous trees N are built on different subsets of data $C_i \subset C$, yielding $\mathbf{X}_i^{C_i \times D}$ such that for each subset, a different tree T_i is built. This random subsampling of data decreases bias, reduces the risk of overfitting the model, and increases the overall generalizability and predictive power of the model Φ. The final model is the collective decision of all trees.

$$\widehat{\mathbf{Y}} = \Phi(\mathbf{X}) = \bigcup_{i=0}^{i=N} T_i(\mathbf{X})$$

The averaging method combines individual decision tree outputs and can be the mean for regression tasks and the mode for classification tasks. If a randomly reduced subset of descriptors $D_i \subset D$ is considered for each tree as proposed by Breiman[6], the method is known as Random Forest.

For boosted trees, initially, a (usually) small decision tree is fit to the entire training data. Iteratively, the errors from previous trees (the strong model $\Phi : \mathbf{X} \mapsto \hat{\mathbf{Y}}$ to step i) are remedied by building the next tree (adding a weak model T_{i+1}) to "fix" those errors at step $i+1$. Instead of fitting the weak learner to (X, Y), it is fit to (X, R_{i+1}).

$$\mathbf{R}_{i+1}^{C\times 1} = -\frac{\partial \mathcal{L}(\Phi_i, \mathbf{X}, \mathbf{Y})}{\partial \hat{\mathbf{Y}}_i}$$

Where the loss function $\mathcal{L}$ can be any differentiable function, e.g., mean squared error $\mathcal{L}(\Phi, \mathbf{Y}, \mathbf{X}) = \frac{1}{C}\sum_C \big(\mathbf{Y} - \Phi(\mathbf{X})\big)^2 = \frac{1}{C}\sum_C \big(\mathbf{Y} - \hat{\mathbf{Y}}\big)^2$ for regression problems or log-likelihood for classification problems. Optimal step size γ is determined by minimizing overall training samples C.

$$\gamma_{i+1} = \arg\min_{\gamma} \mathcal{L}\big(\hat{\mathbf{Y}}_i - \gamma \mathbf{R}_i\big)$$

In addition, a manually controlled (hyperparameter) ν, the shrinkage factor, is employed at each stage to prevent overfitting. Thus, the strong model at each step becomes

$$\Phi_{i+1}(\mathbf{X}) = \Phi_i(\mathbf{X}) - \nu \cdot \gamma_{i+1} \cdot T_{i+1}(\mathbf{R}_{i+1})$$

The iterative nature of the boosting somewhat limits the parallelization of the algorithm, but as the weak learners are trees with a small size, that is not a major concern[7].

Similarly to bagging, a subset of compounds $C_i \subset C$ is used at each stage of building an additional weak learner during boosting, known as stochastic gradient boosting[8].

Descriptors

The exact definitions of the descriptors used in the analysis of the models originate from the Handbook of Molecular Descriptors[9].

X3sol (solvation connectivity index of order 3)

$$X3sol = \frac{1}{2^{3+1}} \cdot \sum_{k=1}^{K} \frac{(\prod_{i=1}^{n} L_i)_k}{(\prod_{i=1}^{n} \delta_i)^{1/2}{}_k}$$

Where all K subgraphs (i.e. molecular fragments) of order 3 (containing exactly 3 atoms) are considered. L is the principal quantum number, δ is the vertex degree (valence number). By definition, fluorine atoms are not included in this calculation as their dimensions are very similar to hydrogen atoms.

VE2_L (average coefficient of the last eigenvector from Laplace matrix)

A Laplacian matrix L is defined atom-pair wise as

$$L_{ij} = \begin{cases} \deg(i), & \text{if } i = j \\ -1, & \text{if } i \neq j \text{ and } i \text{ is adjacent to } j \\ 0, & \text{otherwise} \end{cases}$$

By solving $Lv = \lambda v$, eigenvalue vector λ is obtained. VE2_L is defined as the absolute value of the smallest eigenvalue.

GGI2 (topological charge index of order 2)

Based on the adjacency matrix $A^{N \times N}$ constructed from atoms, where $A_{ij} = 1$ if a bond exists between two atoms and 0 otherwise. Reciprocal square distance matrix D^{-2} defined elementwise $\left[D^{-2}\right]_{ij} = \frac{1}{d_{ij}^2}$, where d is the integer distance of bonds between two atoms.

From their products $\mathbf{M} = \mathbf{AD}$ elements $\mathbf{M}_{ij}$ a charge transfer matrix $\mathbf{CT}$ is derived

$$[\mathbf{CT}]_{ij} = \begin{cases} \delta_{ij} \text{ if } i = j \\ m_{ij} - m_{ji} \text{ if } i \neq j \end{cases}$$

Using its values, only atoms with a distance of 2 from each other are considered, $\delta(\cdot\,,\cdot)$ is the Kronecker delta function equalling 1 if its two arguments are equal and zero otherwise.

$$GGI_2 = \frac{1}{2}\sum_{i}^{A}\sum_{j}^{A}[CT]_{ij} \cdot \delta(2, d_{ij})$$

Eig06_EA(dm) (eigenvalue n. 6 from edge adjacency mat. weighted by dipole moment)

By enumerating all B covalent bonds in a hydrogen-depleted molecular graph, a matrix $\mathbf{E}^{B\times B}$ is produced, where $\mathbf{E}_{ij} = 1$ if two bonds share an atom and 0 otherwise. For dipole weighed edge adjacency matrix, $\mathbf{E}_{ij} = d_j$ if an atom is shared, d_j is the dipole moment of the bond j.

Eig06_AEA(dm) (eigenvalue n. 6 from augmented edge adjacency mat. weighted by dipole moment)

The augmented edge adjacency matrix is defined element-wise as

$$aE = \begin{cases} 1, \text{if } (i,j) \text{ are adjacent} \\ d_{ij}, \text{if } i = j \\ 0, \quad \text{otherwise} \end{cases}$$

GATS4s (Geary autocorrelation of lag 4 weighted by I-state)

I-state is a weighing factor calculated for each atom from properties such as valence electron count δ^{v}, principal quantum number L and sigma electron count δ by

$$I = \frac{\left(\frac{2}{L}\right)^2 \cdot 2 \cdot \delta^{v} + 1}{\delta}$$

It is used as a weighing scheme in counting atom pairs with topological distance 4. Δ_k is the total number of distance 4 atom pairs in a molecule.

$$\text{GATS4s} = \frac{\frac{1}{2\Delta_k}\sum_{i=1}^{A}\sum_{j=1}^{A}\left(I_i - I_j\right)^2 \cdot \delta(4, d_{ij})}{\frac{1}{A-1}\sum_{i=1}^{A}\left(I_i - \text{avg}(I)\right)^2}$$

Figures

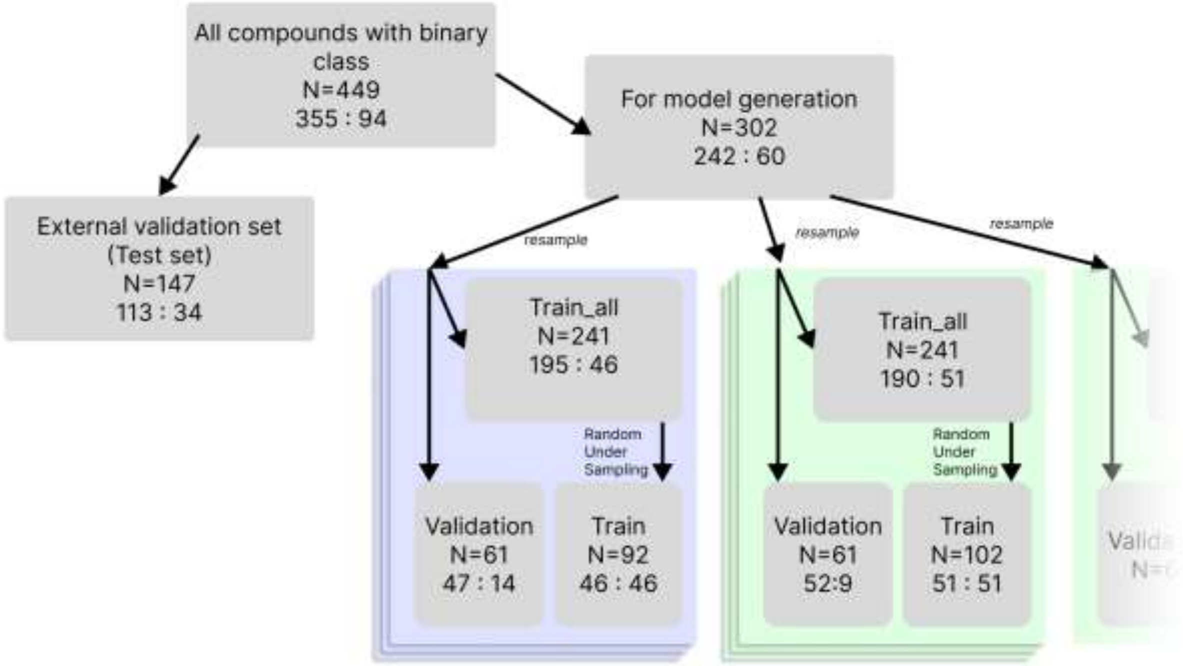

Fig S1. Schema visualizing dataset partitioning after curation and class assignment. To reduce overfitting during model creation, the available dataset of 302 compounds was reshuffled to a new validation set and a new training set after every generation. The training set was again undersampled to balance toxic and nontoxic classes so that the total number of members in each class was equal. The figure also represents models A and B's validation and training sets in the left and right, respectively.

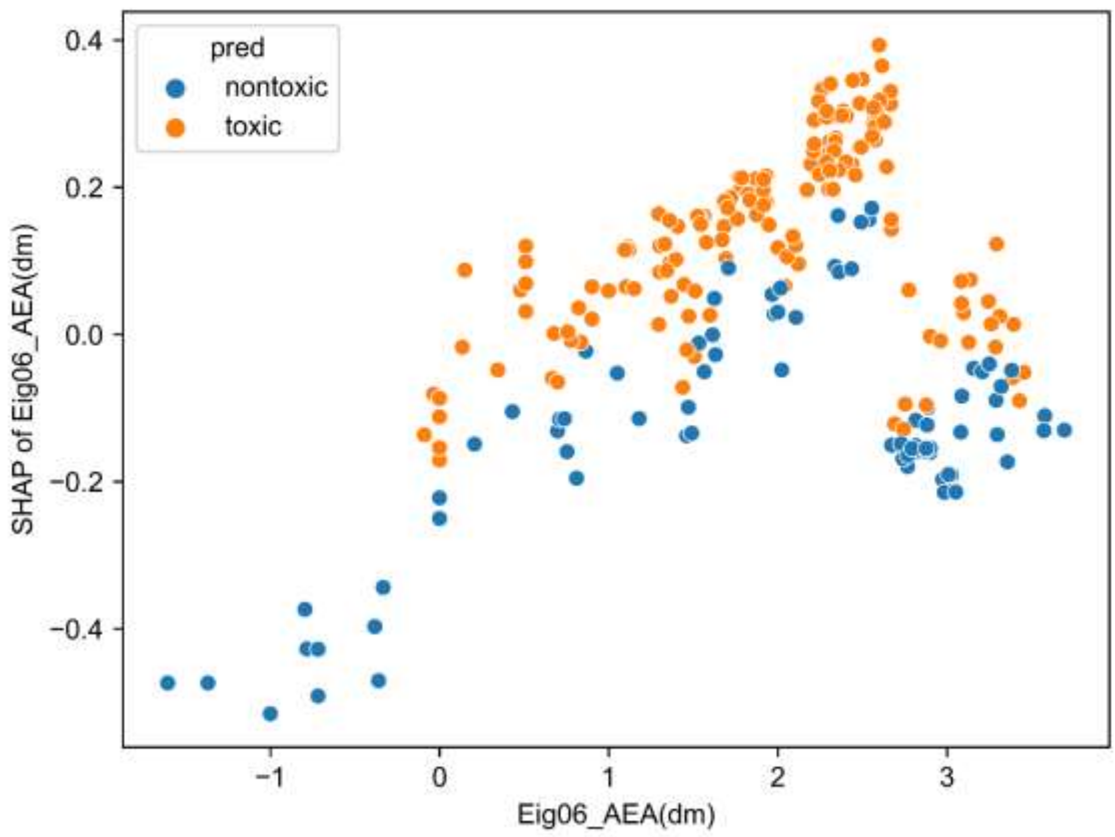

Fig S2. Correlation between Eig09_AEA(dm) SHAP and descriptor values.

Tables

Table S1. Hyperparameter ranges and final values for Model A and Model B.

**Parameter**	**Description**	**Minimum value**	**Maximum value**	**Model A value**	**Model B value**
Shrinkage factor	A regularization parameter for controlling the contribution of each subsequent tree to the overall model.	0.01	0.2	0.0380	0.160
Number of trees	Total number of decision trees included in the ensemble.	91	601	500	231
Fraction of compounds	Proportion of training samples(compounds) used in each iteration of training the ensemble.	0.5	1.0	0.5	0.64
Maximum tree depth	Maximum depth of each decision tree in the ensemble.	3	15	13	15

Cost-complexity regularization parameter	Specifies the extent of post-hoc regularization by simplifying the trees via removing unnecessary node splits.	1E-5	3E-2	6.20E-5	2.12E-3

Table S2. Cross-validation results in test fold on the training set during Bayesian optimization for Model A and Model B.

**Model**	**Sens**	**Spec**	**Acc**	**BalAcc**	**MCC**
Model A	0.607 (0.117)	0.744 (0.112)	0.675 (0.027)	0.651 (0.034)	0.342 (0.055)
Model B	0.723 (0.152)	0.606 (0.225)	0.664 (0.108)	0.637 (0.111)	0.334 (0.212)

References

[1] Sheridan, R. P.; Wang, W. M.; Liaw, A.; Ma, J.; Gifford, E. M. Extreme Gradient Boosting as a Method for Quantitative Structure-Activity Relationships. J. Chem. Inf. Model. **2016**, 56, 2353–2360. https://doi.org/10.1021/ACS.JCIM.6B00591

[2] Kotli, M.; Piir, G.; Maran, U. Pesticide Effect on Earthworm Lethality via Interpretable Machine Learning. J. Hazard. Mater. **2024**, 461, 132577. https://doi.org/10.1016/J.JHAZMAT.2023.132577.

[3] Piir, G.; Sild, S.; Maran, U. Binary and Multi-Class Classification for Androgen Receptor Agonists, Antagonists and Binders. Chemosphere **2021**, 262, 128313. https://doi.org/10.1016/j.chemosphere.2020.128313.

[4] Svetnik, V.; Wang, T.; Tong, C.; Liaw, A.; Sheridan, R. P.; Song, Q. Boosting: An Ensemble Learning Tool for Compound Classification and QSAR Modeling. J. Chem. Inf. Model. **2005**, 45, 786–799. https://doi.org/10.1021/CI0500379/ASSET/IMAGES/LARGE/CI0500379F00006.JPEG.

[5] Breiman, L.; Friedman, J. H.; Olshen, R. A.; Stone, C. J. Classification and Regression Trees; CRC Press, 1984.

[6] Breiman, L. Random Forests. Mach. Learn. 2001, 45, 5–32.

[7] Friedman, J. H. Greedy Function Approximation: A Gradient Boosting Machine. **2001**, 29, 1189–1232. https://doi.org/10.1214/AOS/1013203451.

[8] Friedman, J. H. Stochastic Gradient Boosting. Comput. Stat. Data Anal. **2002**, 38, 367–378. https://doi.org/10.1016/S0167-9473(01)00065-2.

[9] Todeschini, R.; Consonni, V. Handbook of Molecular Descriptors; Methods and Principles in Medicinal Chemistry; Wiley, 2009. https://doi.org/10.1002/9783527628766.
